# PR interval prolongation is significantly associated with aortic root abscess: An age‐ and gender‐matched study

**DOI:** 10.1111/anec.12849

**Published:** 2021-05-03

**Authors:** Utkarsh Kohli, Shirlene Obuobi, Karima Addetia, Takeyoshi Ota, Hemal M. Nayak

**Affiliations:** ^1^ Department of Pediatrics Comer Children's Hospital and Pritzker School of Medicine of the University of Chicago Chicago IL USA; ^2^ Heart & Vascular Center Pritzker School of Medicine of the University of Chicago Chicago IL USA

**Keywords:** aortic root abscess, endocarditis, PR interval prolongation

## Abstract

**Background:**

Electrocardiographic abnormalities, such as PR interval prolongation, have been anecdotally reported in patients with aortic root abscess (ARA). An electrocardiographic marker may be useful in identifying those patients with aortic valve endocarditis who may progress to ARA. The objective of this study is to evaluate the change in the PR interval in patients with surgically confirmed ARA and compare it to age‐ and gender‐matched controls with echocardiographically or surgically confirmed aortic valve endocarditis but without aortic root abscess and those hospitalized with diagnoses other than endocarditis.

**Methods:**

Patients were eligible for enrollment if they were 18 years or older and were hospitalized for either ARA, aortic valve endocarditis, or for unrelated reasons and had at least one 12‐lead electrocardiogram (ECG) prior to or on the day of hospitalization and at least one ECG after hospitalization but prior to any cardiac surgical procedure. Delta PR interval, defined as the difference between the pre‐ and post‐admission PR interval, was the primary outcome of interest. The patients in the ARA group were age‐ and gender‐matched to patients with aortic valve endocarditis and to those without endocarditis. Comparisons of demographic variables and study outcomes were performed.

**Results:**

Eighteen patients with surgically confirmed ARA were enrolled. These patients were age‐ and gender‐matched to 19 patients with aortic valve endocarditis and 18 patients with no past history or evidence of endocarditis during hospitalization. No difference was noted in the baseline PR interval between the groups. However, the PR interval following admission in the aortic root abscess group (201 ± 66 ms) was significantly longer than the PR interval in both the aortic valve endocarditis (162 ± 27 ms) (24%, *p* = .009) and no endocarditis (143 ± 24 ms) (40%, *p* < .001) groups. The primary outcome measure, delta PR interval, was significantly longer in the ARA group (35 ± 51 ms) than no endocarditis (−5 ± 17 ms) (*p* = .001) and aortic valve endocarditis groups (0.2 ± 18) (*p* = .003).

**Conclusions:**

The findings of our study support the notion that the PR interval is more likely to be prolonged in patients with ARA. Since ARA is associated with a high morbidity and mortality, PR interval prolongation in a patient with aortic valve endocarditis should prompt a thorough evaluation for aortic root involvement.

## 1 INTRODUCTION

Aortic root abscess (ARA), which occurs in approximately 20% of patients with aortic valve endocarditis, is associated with a high morbidity and mortality (John et al., 1991; Leontyev et al., 2016; Yang et al., 2020). The risk of developing ARA is higher in patients with aortic paravalvular leaks, mechanical aortic prostheses, aortic valve vegetations, and culture negative endocarditis (Mahmoud et al., 2020). Electrocardiographically, new‐onset high‐grade atrioventricular block has also been associated with a greater likelihood of aortic root involvement in patients with endocarditis (Arnett & Roberts, 1976; Graupner et al., 2002). The mechanistic basis of this finding lies in the close proximity of the cardiac conduction system to the periaortic region. Anecdotal case reports of PR interval prolongation in a handful of patients with ARA (Jain et al., 2015; Kariyanna et al., 2020; Lammers & Dantzig, 2005; Landa et al., 2018) have lent credence to the notion that more subtle conduction abnormalities like PR interval prolongation in patients with aortic valve endocarditis could indicate aortic root involvement. This hypothesis, however, has never been systematically evaluated. The objective of this study is to evaluate the PR interval recorded on 12‐lead electrocardiograms (ECGs) in patients with surgically confirmed ARA and compare it to age‐ and gender‐matched controls with echocardiographically (transesophageal) or surgically confirmed aortic valve endocarditis but without aortic root abscess and those hospitalized with diagnoses other that endocarditis or aortic valve disease.

## 2 METHODS

### 2.1 Study subjects

The study complied with the Declaration of Helsinki and the Institutional Review Board of the University of Chicago Medical Center approved this retrospective study which involved a review of de‐identified medical charts, electrocardiographic tracings, and echocardiographic images. The need for informed consent was waived for this study. Patients were eligible for enrollment if they were 18 years or older and were hospitalized for either ARA, aortic valve endocarditis or for unrelated reasons between January 1, 2000, and December 1, 2020, and had at least one ECG prior to or on the day of hospitalization and at least one ECG after hospitalization but prior to any cardiac surgical procedure. Demographic data, such as age and gender, were collected. Additional data included relevant medical and cardiac surgical history, electrocardiographic findings including heart rate, PR interval, and QRS duration.

### 2.2 Definitions

Aortic Root Abscess Group: Subjects were included in this group if they met the modified Duke criteria (Nishimura et al., 2014) for diagnosis of de‐novo endocarditis, had surgically confirmed aortic root abscess, and had at least one electrocardiogram prior to or on the day of admission, and at least one electrocardiogram following admission but prior to any cardiac surgical procedure.

Aortic Valve Endocarditis Group: Subjects were included in this group if they met the modified Duke criteria (Nishimura et al., 2014) for de‐novo diagnosis of endocarditis, had evidence of aortic valve endocarditis but not aortic root abscess on transesophageal echocardiography (TEE) or during surgery, and had at least one ECG prior to or on the day of admission, and at least one ECG following admission but prior to any cardiac surgical procedure.

No Endocarditis Group: Subjects were included in this group if they had no past history of endocarditis or other aortic valve disease, were admitted with a diagnosis other than endocarditis, and had at least one ECG prior to or on the day of admission and at least one ECG following admission.

### 2.3 Measurements

Measurements were performed using electronic calipers in Muse (GE Healthcare) by cardiac electrophysiologists at the University of Chicago Medical Center. Lead II was preferentially used to calculate the PR interval. If the quality of tracing in lead II was suboptimal, the lead with the best P‐wave morphology was selected. For postadmission electrocardiograms, the longest PR interval was used for analysis in all 3 groups. QRS duration was measured in the lead with the widest QRS. Delta PR interval, defined as the difference between the pre‐ and postadmission PR intervals, was the primary outcome of interest.

### 2.4 Data Analysis and Statistics

The patients in the aortic root abscess group were age‐ and gender‐matched to patients with aortic valve endocarditis and to those with no endocarditis. Data are expressed as mean and standard deviation, or median and interquartile range as appropriate. Since normality assumption was not met, comparisons of demographic variables and study outcomes were performed using nonparametric tests such as Kruskal–Wallis and Mann–Whitney *U* tests for continuous variables and Fisher exact test for categorical variables. *p*‐values of <0.05 were considered significant. All analyses were performed using the statistical software STATA v. 13.0 (StataCorp) and GraphPad Prism v. 9.0. 0 (GraphPad Software).

## 3 RESULTS

### 3.1 Subject characteristics

During the study period, 18 patients with surgically confirmed ARA were enrolled. These patients were age‐ and gender‐matched to 19 patients with aortic valve endocarditis and 18 patients with no past history or evidence of endocarditis during hospitalization. The characteristics of the patients in these 3 groups are shown in Table 1. The patients in the aortic valve endocarditis (95 ± 18 bpm, *p* =.002) and no endocarditis (82 ± 20 bpm, *p* =.03) groups had significantly higher preadmission heart rates than those in the ARA group (75 ± 17 bpm) (Table 1).

**TABLE 1:** Characteristics of the study patients

Parameter	Aortic root abscess (*n* = 18)	Aortic valve endocarditis (*n* = 19)	No endocarditis (*n* = 18)	*p* value
Age (years)	52 ± 15	50 ± 15	52 ± 15	0.79
Gender (Male) (%)	13 (72%)	15 (79%)	13 (72%)	0.86
Baseline heart rate (bpm)	75 ± 17	95 ± 18	82 ± 20	0.005^a^
Heart rate post (bpm)	85 ± 14	91 ± 15	86 ± 22	0.39
Baseline PR interval (ms)	166 ± 29	162 ± 30	148 ± 27	0.14
PR interval post (ms)	201 ± 66	162 ± 27	143 ± 24	0.002^b^
Delta PR interval (ms)	35 ± 51	0.2 ± 18	−5 ± 17	0.03^c^
Baseline QRS duration (ms)	101 ± 16	92 ± 13	89 ± 11	0.03^d^
QRS duration post (ms)	98 ± 12	99 ± 17	85 ± 13	0.02^e^
First degree heart block at baseline (%)	2 (11%)	3 (16%)	1 (6%)	0.68
Advanced heart block (%)	0 (0%)	0 (0%)	0 (0%)	–
Bundle branch block (%)	0 (0%)	1 (5%)	1 (6%)	1.0

^a^ Baseline heart rate in the Aortic Valve Endocarditis group was significantly higher than both Aortic Root Abscess (*p* =.002) and No Endocarditis (*p* =.03) groups.

^b^ PR Interval Post in the Aortic Root Abscess Group was significantly longer than in both Aortic Valve Endocarditis (*p* =.009) and No Endocarditis (*p* =.00) groups.

^c^ Delta PR interval was significantly longer in the Aortic Root Abscess group than No Endocarditis (*p* =.001) and Aortic Valve Endocarditis groups (*p* =.003).

^d^ Baseline QRS duration was longer in the Aortic Valve Abscess group than both Aortic Valve Endocarditis (*p* =.04) and No Endocarditis (*p* =.009) groups.

^e^ The QRS Duration Post was significantly shorter in the No Endocarditis group than both Aortic Valve Abscess (*p* =.008) and Aortic Valve Endocarditis (0.005) groups.

### 3.2 PR intervals

**FIGURE 1:**
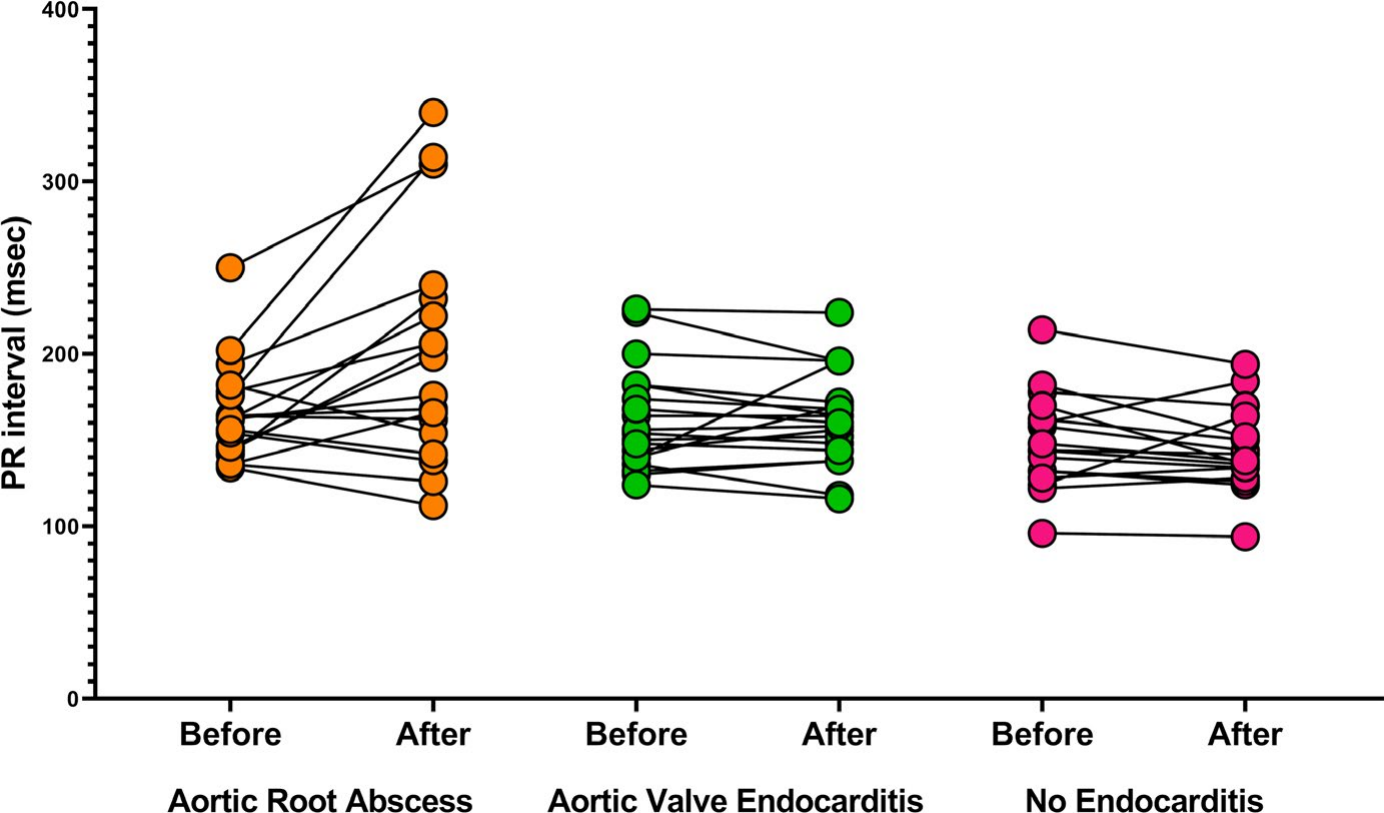
PR interval before and after admission in patients with aortic root abscess, aortic valve endocarditis, and no endocarditis

The baseline electrocardiograms were obtained at a median of 278, 57, 289 days prior to admission in ARA, aortic valve endocarditis, and no endocarditis groups, respectively. No difference was noted in the baseline PR interval between the groups. However, the PR interval following admission in the aortic root abscess group (201 ± 66 ms) was significantly longer than the PR interval in both the aortic valve endocarditis (162 ± 27 ms, *p* =.009) and no endocarditis (143 ± 24 ms, *p* <.001) groups (Figure 1 and Table 1).

### 3.3 Delta PR interval

**FIGURE 2:**
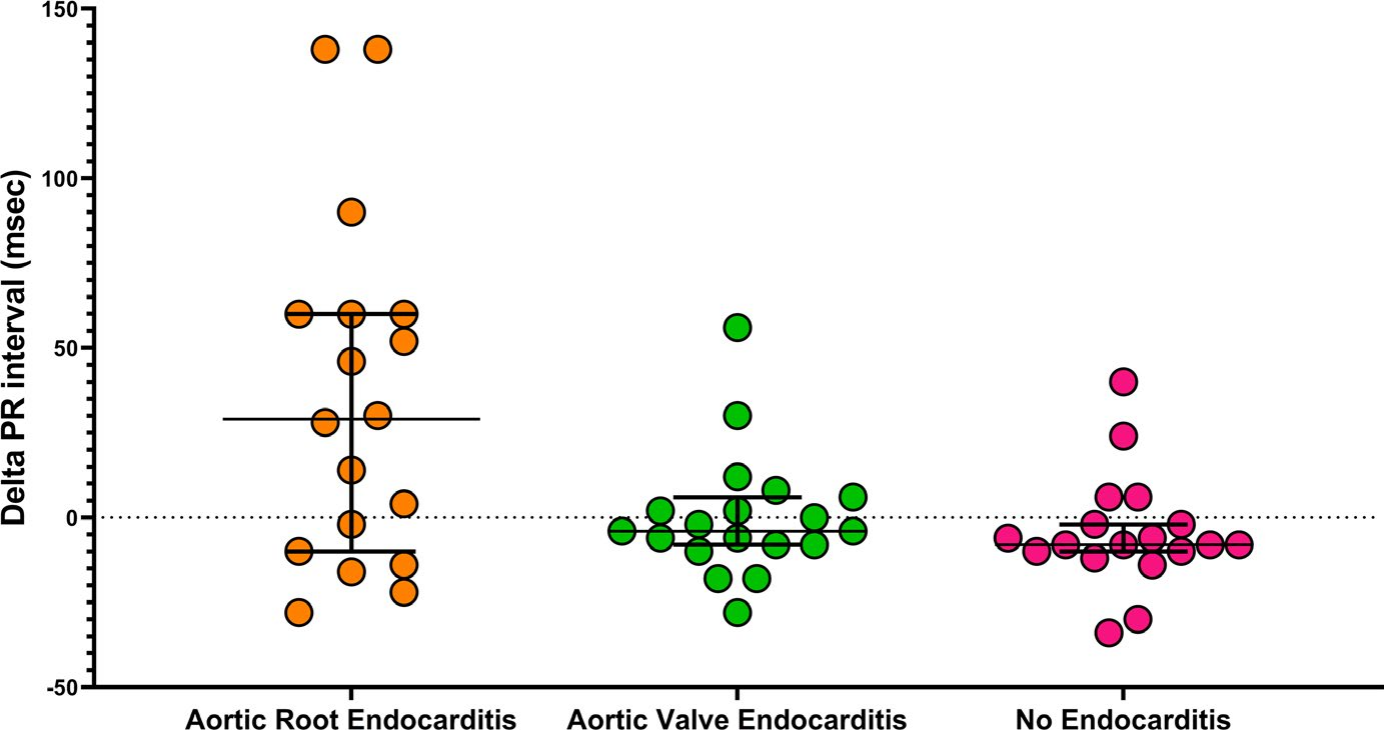
Change in PR interval in patients with aortic root abscess, aortic valve endocarditis, and no endocarditis. Horizontal lines and error bars represent medians and interquartile range, respectively

**FIGURE 3:**
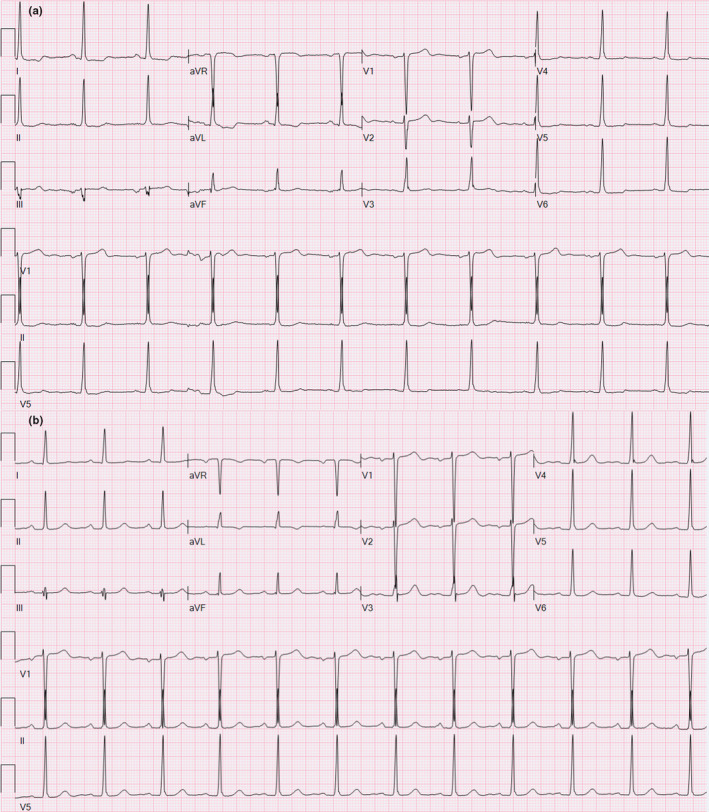
PR interval prolongation from a baseline of 162 ms (Panel A) to 222 ms (Panel B) after diagnosis of aortic root abscess in a 74‐year‐old female

The primary outcome measure, the delta PR interval, was significantly longer in the aortic root abscess group (35 ± 51 ms) than no endocarditis (−5 ± 17 ms, *p* =.001) and aortic valve endocarditis groups (0.2 ± 18 ms, *p* =.003) (Figures 2 and 3 and Table 1).

## 4 DISCUSSION

To the best of our knowledge, this is the first study to systematically evaluate the change in the PR interval in patients with ARA in comparison with age‐ and gender‐matched controls with aortic valve endocarditis without aortic root involvement and those without a diagnosis of endocarditis. The main finding of this study is that the patients with ARA show a greater prolongation in the PR interval than patients with aortic valve endocarditis.

These findings imply that in patients with aortic valve endocarditis, prolongation of PR interval could be indicative of aortic root involvement and should prompt a thorough evaluation for the same. The timely diagnosis of ARA is crucial to reducing morbidity and mortality and continues to remain a challenge. Transesophageal echocardiography (TEE) has been shown to be more sensitive than transthoracic echocardiography (TTE) and its utilization early during the course of the illness can help hasten the diagnosis (Leung et al., 1994). A recent study showed ECG‐gated CT to have similar sensitivity to TEE suggesting its role in situations where TEE is contraindicated or not available (Ye et al., 2020).

Despite surgical intervention, several large studies have noted a high morbidity and mortality in patients with ARA (John et al., 1991; Leontyev et al., 2016; Yang et al., 2020). An overall mortality of 36%, with a 30‐day mortality of 13% and 120‐day mortality of 16% has been reported (Croon et al., 2020).

The location of the AV node in human hearts is variable. Approximately 50% of humans have a predominantly right‐sided AV node, 30% have a left‐sided orientation, and the remaining 20% have an AV node running under the membranous septum just below the endocardium. The latter two anatomic variants are thought to predispose patients to increased risk of conduction abnormalities following transcatheter aortic valve replacement (Judson et al., 2019; Kawashima & Sato, 2014). In addition to the extent and precise site of aortic root involvement, these anatomic variations might underlie the variability in PR prolongation in patients with ARA.

Our study had several limitations. The sample size was small but reflected the fact that only patients with surgically confirmed ARA were included in the study group. This was done to minimize misclassification and bias. Conduction abnormalities are common after cardiac surgical procedures; therefore, only patients with postadmission ECGs prior to a cardiac surgical procedure were included in the study. Furthermore, all electrocardiographic measurements were performed using electronic calipers by cardiac electrophysiologists to minimize error in calculation of intervals. We adjusted our analysis for known confounders such as age and gender by matching. However, the possibility of confounders which may have been present but were not adjusted for cannot be eliminated.

In conclusion, the findings of our study support the notion that the PR interval is more likely to be prolonged in patients with ARA versus those with aortic valve endocarditis alone. Since aortic root abscess is associated with a high morbidity and mortality, PR interval prolongation in a patient with aortic valve endocarditis should prompt a thorough evaluation for aortic root involvement.

## CONFLICT OF INTEREST

None.

## AUTHOR CONTRIBUTIONS

Drs. Kohli and Obuobi were responsible for collecting the data. Drs. Addetia and Ota were responsible for collecting the data and critically editing the manuscript. Drs. Kohli and Nayak were responsible for writing the manuscript. Dr. Nayak was responsible for the study concept, design and overall completion of the project.

## ETHICAL APPROVAL

The study conforms to the US Federal policy for protection of human subjects and the study complied with the Declaration of Helsinki and the Institutional Review Board of the University of Chicago Medical Center approved this retrospective study. The need for informed consent was waived for this study.

## Data Availability

The data that support the findings of this study are available from the corresponding author upon reasonable request.
